# Reducing central line-associated bloodstream infections (CLABSI) by improved identification of primary infection sites through preliminary blood culture notifications

**DOI:** 10.1017/ash.2024.442

**Published:** 2024-10-17

**Authors:** Werner E. Bischoff, Cheryl Kieta, Tiffany LaFontaine, Corrianne Billings

**Affiliations:** 1 Internal Medicine/Section on Infectious Diseases, Wake Forest University School of Medicine, Winston Salem, NC, USA; 2 Infection Prevention and Health System Epidemiology, Atrium Health Wake Forest Baptist, Winston Salem, NC, USA

## Abstract

Central line-associated bloodstream infections (CLABSI) pose a significant patient risk. To address incorrect classifications of infectious events as CLABSI, the surveillance process was changed from using finalized blood culture to preliminary blood culture results as trigger of timely, in-depth reviews. This improved the detection of true CLABSI events.

## Methods

A quasi-experimental study was conducted from 04/01/2022 to 09/30/2023 to improve the accuracy of CLABSI identification through extending the infection detection window by changing from daily checking of final blood culture results to evaluating all preliminary blood culture results within 24 hours. Using the earlier identification of potential events allowed the IP team to consult with the clinical teams regarding primary sites of infections other than central lines that would have not been addressed pre-intervention. We compared the observed CLABSI rates and standardized infection ratios (SIR) with the uncorrected rates and SIR accounting for the avoided events.

**Figure 1.**
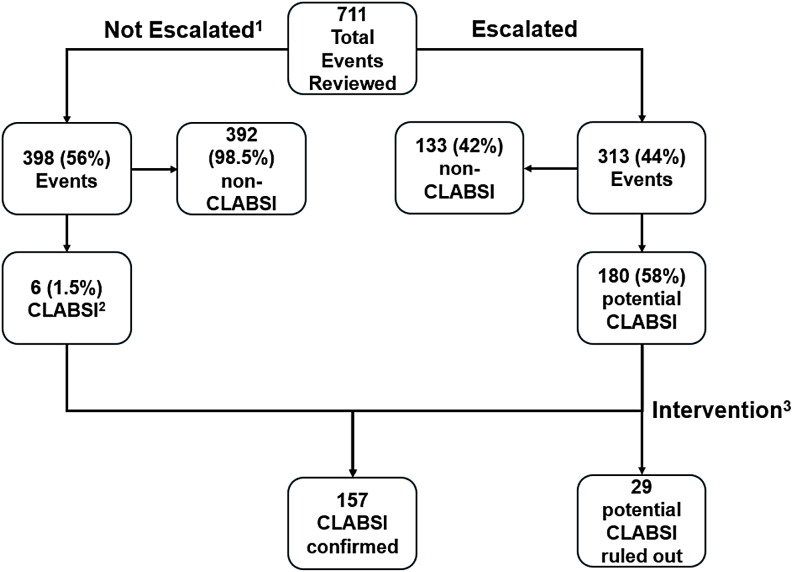
Event review flowchart. 1. Non-Escalation Criteria: Primary site other than Central Line established. One common commensal only, no growth in other cultures. Known exclusion criteria such as ventricular assist device (VAD), extracorporeal membrane oxygenation (ECMO), or confirmed patient self-injection. Mucosal barrier injury (MBI) criteria met. Repeat culture within a repeat infection timeframe (RIT) of a present on admission infection (POA) culture. No central line present at time of event. 2. CLABSI criteria in non-escalated events: No opportunity for imaging or documentation due to: Infection Window Period elapsed. Patient expired. Patient discharged. 3. Intervention elements: Imaging studies – ensure warranted imaging studies are performed in a timely manner to align with infection window period. Clinical correlation – documentation of provider initiation or continuation of treatment for primary site of infection, often pneumonia or intraabdominal infection. Timely culture collection of purulent sites suspected to be source of bacteremia. Documentation/clarification of abnormal/changes in wound characteristics, signs and symptoms, line access, or patient line injection.

The study included all inpatients with central venous catheters (CVC, total Central Line days: 115,670) and at least one blood culture drawn during admission to five acute care hospitals in North Carolina. The hospitals are a tertiary care academic medical center (885 licensed beds) including a children’s hospital (155 beds), a mid-size community hospital (350 beds), and three smaller community hospitals (50, 94 and 130 beds). Surveillance infection preventionists (SIP) evaluated all preliminary blood culture results within 24 hours by using National Healthcare Safety Network (NHSN) definitions.^2^ The SIP team consisted of three IPs certified in Infection Control (Certification Board of Infection Control and Epidemiology, Inc. [CBIC®], Arlington, VA). Preliminary blood culture results included Gram stains or blood culture identification panel (BIOFIRE® Blood Culture Identification Panel, BioMerieux, Lyon, France) through an automated notification system using NHSN bacteremia criteria (EPIC Bugsy, EPIC, Verona, WI). SIP assigned cases to the following groups: (1) not escalated for CLABSI investigation (exclusion criteria listed in Figure 1) or (2) escalated to IP team for further review. IP team members reached out to the medical teams to identify potential primary site infections and discuss the need for further testing and documentation to classify bacteremia as secondary. The Medical Director of IP reviewed the cases on a regular basis and supported the discussions with the clinical teams. A subset of events was used for a time/effort assessment. Clopper-Pearson exact binomial tests were used to compare CLABSI burden as the outcome measure with and without the intervention.

## Results

Preliminary blood culture results in a total of 711 events were reviewed (Figure 1). Of those, 398 (56%) were dismissed as not eligible for CLABSI investigation, 313 (44%) were escalated to the IP team with 133 (42%) determined to be non-CLABSI, and 180 (58%) referred to further assessment by IP and clinical teams. A primary cause other than CVC was established in 29 events leading to a reduction in potential CLABSI cases from 186 to 157 (15.6%; *P* <.0001) and a decrease in CLABSI rates from 1.608 to 1.357/1,000 CL days for all units (Table 1). In publicly reported units CLABSI cases were reduced from 110 to 93 (15.5%; *P* <.0001) and 1.613/1,000 CL days to 1.363/1,000 reduction in CL days. This also led to a reduction in SIR (*P* <.0001). Of the 29 events six were associated with Methicillin-resistant *Staphylococcus aureus* (MRSA), six with *Pseudomonas aeruginosa*, three each with *Staphylococcus epidermidis* and *Serratia marcescens*, two each with *Klebsiella pneumoniae*, *Morganella morganii,* and Yeast followed by detection of a single pathogen (table in supplement). During the trial, the number of bacteremia/patient with central lines remained constant.

**Table 1.** Impact of preliminary blood culture notifications on CLABSI event detection (rates and SIR^*^)

	Number of Primary Site Infections Identified other than CLABSI (Uncorrected vs Actual Events)	Uncorrected Rate/1,000 Central Line Days; Uncorrected SIR^*^	Actual Rate/1,000 Central Line Days; Actual SIR^*^	Percent Rate and SIR^*^ Reduction	Statistical Analysis^**^ (*P* value; 95% Confidence Interval)
All Units (n = 73)	29 (186 vs. 157)	1.608; 1.481	1.357; 1.250	–15.6	<0.0001; –21.6, –10.7
Publicly Reportable Units^***^ (n = 33)	17 (110 vs. 93)	1.613; 1.477	1.363; 1.248	–15.5	<0.0001; –23.6, –9.3

* SIR, standardized infection ratio.

** Clopper–Pearson exact binomial test.

*** Centers for Medicare & Medicaid Services (CMS) unit selection based on CDC/NHSN patient care location mapping.

Interventions established the following primary causes of bacteremia (table in supplement): pneumonia (12, 41%), burn (6, 21%), endocarditis (3, 7% [one patient had an additional skin infection]), patient line injection (2, 7%), intraabdominal infection (2, 7%), skin infection (2, 7%), bone/osteomyelitis (1, 3%), central line documentation clarification (1, 3%), and necrotizing enterocolitis (1, 3%). Pathogens in the two leading primary sites included *Pseudomonas aeruginosa* (n = 5) and *Serratia marcescens* (n = 3) in pneumonia and MRSA (n = 4) in burn infections. The pathogen distribution matched the findings in the confirmed CLABSI group except for lower *Pseudomonas aeruginosa* counts in the latter. Interventions included imaging studies (n = 11), clinical correlation (provider initiated or continued treatment specifically for pneumonia, n = 6), culture collection of purulent sites (n = 1), and documentation/clarification of abnormal/changes in wound characteristics, signs and symptoms, line access, or patient line injection (n = 10).

A time/effort study was conducted in 39 (12%) of 313 escalated events to assess the additional burden. The SIP team spent an average of 15.6 minutes per case. 1.9 caregivers were contacted by IP with 15 minutes each spent on discussing the case and 25.6 minutes spent on follow-up activities such as providing additional information, education, and chart documentation. The total time commitment was 56.2 minutes/case.

## Conclusions

Accurate and timely identification is paramount for reducing CLABSI.^1,2^ NHSN provides detailed criteria to identify CLABSI.^3^ Special emphasis is placed on confirming the primary nature of a bloodstream infection before proceeding. Our study targeted the early identification phase marked by the report of preliminary blood culture results such as a Gram stain or blood culture identification panel to ensure timely assessment of the true infection sources. This led to a significant improvement in primary infection site identification other than CVC within the narrow infection window timeframes prescribed by NHSN definitions.

CLABSI events serve as an important quality outcome measure for regulatory and reputational agencies. As defined by NHSN, the Centers for Medicare and Medicaid Services (CMS) and private insurers use this measure in pay-for-performance programs.^4^ The prevalence of CLABSI has decreased over several decades even with the recent uptick during the SARS-CoV-2 pandemic placing greater emphasis on single events.^5^ However, there are concerns that NHSN definitions are subjective and do not reflect the true performance of the respective institutions, although criteria has been updated since this study.^6^ Studies have shown the potential for overestimating of CLABSI burden by up to 30% driven by misclassification of the primary sites of infection due to gaps in the definitions or missed chances for clarification by the medical teams.^7^ For example, pneumonia was most often cited as primary site of infection in our study as it was by Al Hammadi *et al.* ^7^ Hsueh et al. further differentiated between end-of-life CLABSI, definition-based CLABSI (non-preventable) and preventable CLABSI events.^8^ Definition-based CLABSI most closely matched our approach with 18.4%. Broadening the window of opportunity for correction by using the first available sign of a potential CLABSI event allowed us to reassign approximately 15% of events as non-CLABSI.

Our study has limitations. The case scenarios and time estimates may be unique to our clinical practice. During the trial IPs participated in medical team rounds in intensive care settings to share information pertinent to device related infections such as need for and duration of device use. This reduced the overall risk of CLABSI but did not alter the primary objective of improving the accuracy of CLABSI detection. In addition, CLABSI rates and total bacteremia counts per patient remained stable during the trial.

There is an ongoing discussion of the value of the CDC/NHSN definitions for CLABSI as an indicator of the quality of care.^9^ Misclassified CLABSI present a false image of performance, distract from preventable events, and reduce confidence in overall surveillance efforts. Moving the process of CLABSI identification from finalized blood cultures to preliminary blood culture results provided an insight into the extend of misclassification subsequently improving the accuracy of CLABSI detection.

## Supporting information

Bischoff et al. supplementary material Bischoff et al. supplementary material

## Supplementary material

For supplementary material accompanying this paper visit http://doi.org/10.1017/ash.2024.442. click here to view supplementary material
