# Supplementary material for: Reducing central line-associated bloodstream infections (CLABSI) by improved identification of primary infection sites through preliminary blood culture notifications
Source: Antimicrob Steward Healthc Epidemiol. 2024 Oct 17;4(1):e178. doi: 10.1017/ash.2024.442 (PMC11500242; doi:10.1017/ash.2024.442)
Supplement: Bischoff et al. supplementary material [file S2732494X2400442Xsup001.docx]

Supplement:

Table: Patient Characteristics of the 29 non-CLABSI Events

| **Patient** | **Facility** | **Age** | **Gender** | **Race** | **Ethnicity** | **ICU vs non-ICU** | **Medical vs Surgical Services** | **Service Line** | **Primary Diagnosis** | **Primary Site of Infection (corrected)** | **Organism(s)** | **Intervention** |
| --- | --- | --- | --- | --- | --- | --- | --- | --- | --- | --- | --- | --- |
| 1 | Small-size Community Hospital | 34 | Female | White | Non-Hispanic | Non-ICU | Medical | Medicine | Endocarditis | Endocarditis | Veionella, Gram-negative rods | Imaging Studies |
| 2 | Academic Medical Center | 43 | Male | Asian | Non-Hispanic | ICU | Medical | Burn | Burn | Burn | MRSA | Documentation of Wound Condition |
| 3 | Academic Medical Center | 52 | Female | White | Non-Hispanic | ICU | Medical | Medicine | Cardiogenic shock | Pneumonia | Pseudomonas aeruginosa | Imaging Studies |
| 4 | Small-size Community Hospital | 43 | Male | White | Non-Hispanic | Non-ICU | Medical | Medicine | Endocarditis | Injection | Morganella morganii, Klebsiella pneumoniae | Documentation of Patient Line Injection |
| 5 | Academic Medical Center | 56 | Male | White | Non-Hispanic | ICU | Medical | Cardiovascular ICU | Precordial chest pain | Pneumonia | MRSA | Imaging Studies |
| 6 | Academic Medical Center | 37 | Male | White | Non-Hispanic | ICU | Medical | Burn | Burn | Burn | Pseudomonas aeruginosa, Staphylococcus aureus | Documentation of Wound Condition |
| 7 | Academic Medical Center | <1 | Female | Black | Non-Hispanic | ICU | Medical | Neonatology | Premature infant of 29 weeks gestation | Skin | MRSA | Documentation of Signs & Symptoms |
| 8 | Mid-size Community Hospital | 30 | Female | White | Non-Hispanic | Non-ICU | Medical | Hospitalist | Bacteremia | Endocarditis | Yeast | Imaging Studies |
| 9 | Academic Medical Center | <1 | Male | Asian | Non-Hispanic | ICU | Medical | Neonatology | Stage III necrotizing enterocolitis in newborn | Necrotizing enterocolitis | Klebsiella variicola | Documentation of Signs & Symptoms |
| 10 | Academic Medical Center | 22 | Female | White | Non-Hispanic | Non-ICU | Medical | Burn | Burn | Burn | MRSA | Documentation of Signs & Symptoms |
| 11 | Academic Medical Center | 22 | Female | White | Non-Hispanic | Non-ICU | Medical | Burn | Burn | Burn | Yeast | Documentation of Signs & Symptoms |
| 12 | Academic Medical Center | 62 | Female | White | Non-Hispanic | Non-ICU | Medical | Medicine | Shock | Central Line documentation clarification | Morganella morganii | Line Access Documentation |
| 13 | Academic Medical Center | 64 | Male | White | Non-Hispanic | Non-ICU | Medical | Hematology | Acute Myeloid Leukemia | Pneumonia | E. coli | Imaging Studies |
| 14 | Academic Medical Center | 63 | Male | White | Non-Hispanic | Non-ICU | Medical | Burn | Burn | Burn | MRSA | Documentation of Signs & Symptoms |
| 15 | Mid-size Community Hospital | 81 | Male | White | Non-Hispanic | ICU | Medical | Medicine | Neutropenic fever | Pneumonia | Pseudomonas aeruginosa | Imaging Studies |
| 16 | Academic Medical Center | 28 | Female | White | Non-Hispanic | Non-ICU | Medical | Hospitalist | Myalgia | Injection | Staphylococcus epidermidis, Burkholderia cepacia | Documentation of Patient Line Injection |
| 17 | Academic Medical Center | 32 | Male | Black | Non-Hispanic | ICU | Medical | Trauma ICU | Trauma | Intraabdominal Surgical Site Infection | Candida albicans | Primary Site Culture |
| 18 | Academic Medical Center | 25 | Male | Other | HLS | Non-ICU | Medical | Burn | Burn | Burn | MRSA | Documentation of Signs & Symptoms |
| 19 | Academic Medical Center | 62 | Male | Black | Non-Hispanic | ICU | Surgical | Renal Transplant | Pre-evaluation for kidney transplant | Pneumonia | Serratia marcescens | Clinical Correlation |
| 20 | Academic Medical Center | 44 | Female | White | Non-Hispanic | Non-ICU | Medical | Bariatric/Minimally Invasive Surgery | Severe protein-calorie malnutrition | Pneumonia | Serratia marcescens | Imaging Studies |
| 21 | Academic Medical Center | 70 | Female | White | Non-Hispanic | ICU | Medical | Medicine | Septic shock | Pneumonia | Acinetobacter species | Clinical Correlation |
| 22 | Academic Medical Center | 0 | Male | Other | Hispanic | ICU | Medical | Neonatology | Extreme immaturity of newborn, gestational age 24 completed weeks | Pneumonia | Serratia marcescens | Imaging Studies |
| 23 | Academic Medical Center | 55 | Male | Other | Hispanic | ICU | Medical | Medicine | Acute Myeloid Leukemia | Pneumonia | Pseudomonas aeruginosa | Imaging Studies |
| 24 | Academic Medical Center | 49 | Female | White | Non-Hispanic | Non-ICU | Medical | Oncology Surgery | Metastatic colon cancer to liver | Intraabdominal Surgical Site Infection | Staphylococcus epidermidis, Streptococcus anginosus | Clinical Correlation |
| 25 | Mid-size Community Hospital | 69 | Female | White | Non-Hispanic | Non-ICU | Medical | Hospitalist | Shock | Endocarditis and Skin Infection | Staphylococcus aureus, Enterococcus faecium | Clinical Correlation |
| 26 | Academic Medical Center | 84 | Female | Black | Non-Hispanic | ICU | Surgical | Cardiothoracic Surgery | Dissection of ascending aorta | Pneumonia | Pseudomonas aeruginosa | Clinical Correlation |
| 27 | Academic Medical Center | 49 | Male | Black | Non-Hispanic | Non-ICU | Medical | Hospitalist | Osteomyelitis right foot | Bone - Osteomyelitis | Klebsiella pneumoniae | Imaging Studies |
| 28 | Academic Medical Center | 55 | Male | Black | Non-Hispanic | ICU | Medical | Neurology Stroke | Cerebrovascular accident (CVA) | Pneumonia | Bacillus cereus, Staphylococcus epidermidis, Staphylococcus hominis | Imaging Studies |
| 29 | Academic Medical Center | 35 | Male | White | Non-Hispanic | ICU | Surgical | Cardiothoracic surgery | Mitral valve stenosis, severe | Pneumonia | Pseudomonas aeruginosa | Clinical Correlation |
